# Lower Limb Loading during Gait in Patients Long Period after Total Hip Arthroplasty Revision

**DOI:** 10.1155/2016/7538236

**Published:** 2016-08-04

**Authors:** Eliska Kubonova, Zdenek Svoboda, Miroslav Janura, Jiri Gallo, Sarka Duskova

**Affiliations:** ^1^Department of Natural Sciences in Kinanthropology, Faculty of Physical Culture, Palacký University Olomouc, Třída Míru 117, 77111 Olomouc, Czech Republic; ^2^Department of Orthopaedics, Faculty of Medicine and Dentistry, Palacký University Olomouc, Faculty Hospital, I.P. Pavlova 6, 77900 Olomouc, Czech Republic; ^3^Department of Rehabilitation, Faculty of Medicine, University of Ostrava, Syllabova 19, 70300 Ostrava, Czech Republic

## Abstract

The aim of the study was to assess lower limb loading during walking after unilateral total hip arthroplasty (THA) revision. Twenty-three THA revision subjects (12 men, 11 women) were divided into three groups according to time since surgery as 1 to 6 years, 6 to 11 years, and over 11 years. Two force plates were used to measure the ground reaction force during the stance phase. On the operated limb, compared to nonoperated limb, we found lower first vertical peak in the group of 1 to 6 years after revision and lower propulsion peak in the group of 6 to 11 years since revision. In the group of 11 years since THA revision, no significant difference was found. With advancing years after surgery, the stance phase duration got reduced and propulsion peak increased in the operated limb; minimal vertical force decreased and the time of minimal vertical force increased in the nonoperated limb. The study findings suggest the tendency to a more gradual and safer weight acceptance on the operated limb during the first years after THA revision, followed by limitation of foot propulsion. Despite this fact, lower limb loading can be considered as symmetrical across the whole measured period.

## 1. Introduction

Total hip arthroplasty (THA) is a huge intervention into the human locomotor system, and the results of complex therapeutic care are strong for most patients [1]. Research in this field has examined postoperative conditions of a patient's musculoskeletal system undergoing primary total hip arthroplasty (quantitatively and qualitatively) from 3–6 weeks after surgery [2, 3], to 2–6 months after surgery [4, 5], to the most frequently used term of one year after surgery [6–9].

It was shown that THA patients' gait mechanics do not return to normal until one year after surgery. The differences between gait performance of THA subjects compared to gait performance of normal population include decreased gait speed [5], kinematic adaptations at the ankle of the operated limb, hip of the nonoperated [6], thorax and pelvis [3], and kinetic adaptations such as decreased hip extension and abductions force moments [5]. Similarly, asymmetrical limb loading persists after unilateral hip replacement surgery [4].

The average age of patients undergoing THA is decreasing [10], which is related to the increase in the number of patients undergoing THA revision and the reduction in their average age [10, 11]. These patients are an active population in most cases. The current scientific literature contains articles about THA revision that focus more on surgical techniques than on the clinical implications (gait kinetics or kinematics) and the latter, where present, are limited to questionnaires on the quality of life and the amount of physical activity during the postoperative period [10, 12]. What is lacking is substantiated information on the efficacy of physical activities in patients after THA revision (especially walking) and the nature of the changes that occur several years after surgery. In this context, the paper herein attempts to address the increasing demand for objective information on the health condition following THA revision.

Patients undergoing THA revision represent a very specific group due to previous experiences with the primary implant and later also with other structural defects leading to second-time joint implant [1, 13]. These patients suffer from more limitations to the quality of their lives with compromised extent and intensity of physical activity when compared to patients after primary THA [12].

The hip joint, as a bearing joint in man's motor scheme, plays a very important part in mobility. In THA patients, a certain degree of load asymmetry is expected pursuant to joint reimplant. If the patient has the tendency of avoiding putting load on the operated limb or if there are some functional limitations in the concerned limb, these features are then reflected in the ground reaction force (GRF) values [8] and in their variability [14]. As an important implication here, we also consider the association of this possible asymmetry to other parts of the musculoskeletal system (e.g., low back pain) and the asymmetric strength results of the lower limbs [15]. Therefore, we analysed the ground reaction forces during walking in patients following THA revision and observed the long-term changes in the behaviour of these forces after the surgery. Multiannual research is absent in this field, and it is not clear how THA revision affects movement activities several years after surgery. Our study assessed the influence of the postoperative period on operated and nonoperated lower limb loading in individuals with unilateral THA revision during walking. We hypothesize that lower limb loading and its asymmetry differ between groups with various period after THA revision.

## 2. Materials and Methods

### 2.1. Subjects

Twenty-three subjects who underwent total hip unilateral arthroplasty revision (12 men, 11 women, from 1 to 16 years after THA, with age 59.5 SD (standard deviation) 7.5 years, height 168.2 SD 9.5 cm, weight 79.2 SD 16.9 kg, and time after surgery 7.5 SD 4.4 years) participated in this study. The participants were recruited from the university hospital database. All subjects were introduced with experiment and signed informed consent. Design of the study was approved by local Ethical Committee.

At least one year had passed since unilateral THA revision. A transgluteal approach, with release of the anterior third of the gluteus medius muscle from the tip of the greater trochanter, was applied in all subjects. Subjects walked without a walking aid or pain. Subjects with other joint disorders or other primary or revision total arthroplasty (knee, hip) were excluded.

Twenty-three enrolled subjects were distributed into three groups depending on the postsurgery duration:

(i) *One to 6 years* (1.0–6.0 years, mean: 2.3, SD 1.5 years) after THA revision, *n* = 9 (age 60.1 SD 6.5 years, height 172.4 SD 9.9 cm, weight 85.7 SD 16.3 kg, and time after surgery 2.3 SD 1.5 years).
(ii) *Six to 11 years* (6.0–11.0 years, mean: 8,9, SD 1.1 years) after THA revision, *n* = 7 (age 63.9 SD 4.0 years, height 164.0 SD 6.8 cm, weight 75.6 SD 15.2 kg, and time after surgery 8.9 SD 1.1 years).
(iii) *Over 11 years* (over 11.0 years, mean: 12.7, SD 1.6 years) after THA revision, *n* = 7 (age 54.3 SD 9.0 years, height 167.0 SD 12.1 cm, weight 74.4 SD 20.6 kg, and time after surgery 12.7 SD 1.6 years).

### 2.2. Procedures

Subjects were asked to walk naturally barefoot on a 10-meter-long sidewalk. Two force plates (Kistler 9286AA, measuring frequency 200 Hz, Kistler Instrumente AG, Winterthur, Switzerland) were used within the wooden sidewalk to measure GRF during the stance phase. They were placed approximately in the middle of the 10-meter-long sidewalk. Overall, 10 gait trials were measured, and the last five successful gait trials of each subject were evaluated. As successful we considered a trial, when subject touches the force plates by both limbs (one plate for each limb) without targeting.

### 2.3. Data Processing

**Figure 1:**
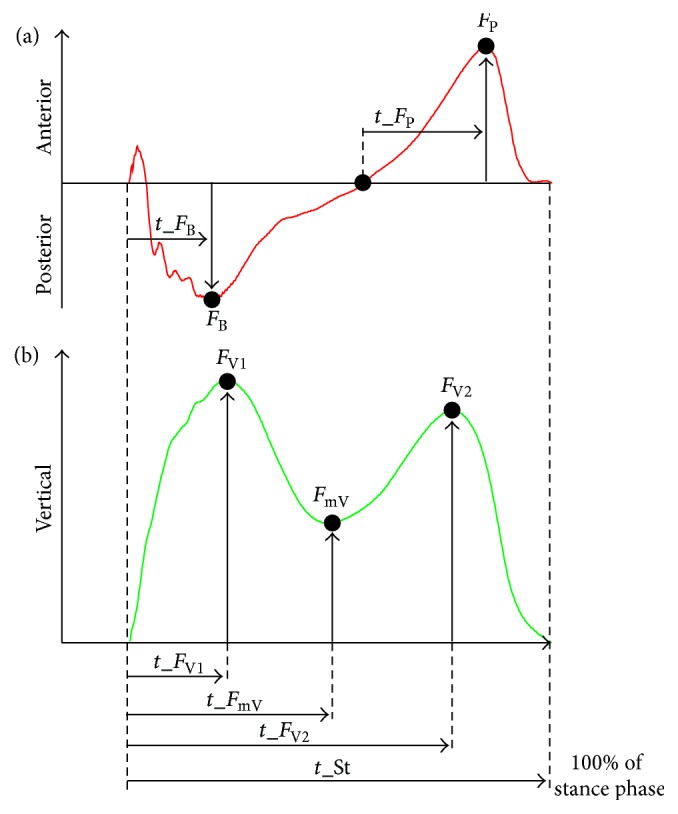
Variables of anterior-posterior (a) and vertical components (b) of GRF. *t*_St: stance phase duration, *t*_*F*_B_: time of breaking peak, *t*_*F*_P_: time of propulsion peak, *t*_*F*_V1_: time of the first vertical peak, *t*_*F*_mV_: time of minimal vertical force, *t*_*F*_V2_: time of the second vertical peak, *F*_B_: breaking peak, *F*_P_: propulsion peak, *F*_V1_: first vertical peak, *F*_V2_: second vertical peak, and *F*_mV_: minimal vertical force.

The data was filtered applying the 4th-order Butterworth low pass filter with the cut-off frequency 30 Hz. In our study, we focused on the vertical and anterior-posterior components of GRF. The vertical GRF component represents, in particular, the weight-bearing function of the limb together with other variables derived from the anterior-posterior GRF component; it can also help in assessing the gait style [16]. The medial-lateral GRF component was excluded from our study due to insufficient intertrial reliability [17]. Force behaviour derived from force peaks, timing, and force impulses [18] meant possible variables for analysis. For proper comprehension, we opted for only peak values of the vertical and anterior-posterior components and their timing (Figure 1). Force variables were normalized to body weight; temporal variables were normalized to stance phase duration.

For assessing symmetry between the limbs, we used the equation presented by Robinson et al. [19]: symmetry index (SI) = (nonoperated − operated)/((nonoperated + operated)/2)*∗*100%. A positive value means higher value of variable on the nonoperated limb; a negative value means higher value of variable on the operated limb.

### 2.4. Statistical Analysis

Statistical analysis was performed using Statistica version 12.0 (StatSoft Inc., Tulsa, OK, USA). Normal data distribution was assessed by Shapiro-Wilk test. The results of the test showed nonnormal data distribution for most of variables, and thus nonparametric procedures were used. Comparisons of mean values of variables on operated and nonoperated limbs were performed using Wilcoxon test. Differences between groups were assessed using Mann-Whitney *U* tests. *p* values less than 0.05 were considered significant. The effect size was determined by *r* = *Z*/√*N*, where *Z* was the standardized value of the Mann-Whitney *U* test and *N* was the total number of samples. The effect size was considered small when it is 0.1 ≤ *r* < 0.3, medium when it is 0.3 ≤ *r* < 0.5, and large when it is *r* ≥ 0.5 [20].

## 3. Results

Walking velocity was comparable (no significant differences) in all three observed groups (1 to 6 years, 0.94 ± 0.10 m·s^−1^, 6 to 11 years, 0.98 ± 0.06 m·s^−1^, and over 11 years, 0.96 ± 0.13 m·s^−1^). The observed GRF values from the data are presented in Table 1 and the assessment of symmetry is shown in Table 2.

### 3.1. Operated versus Nonoperated Lower Limbs

Higher values of the first vertical peak (*p* = 0.015) and shorter time of the first vertical peak (*p* = 0.015) were found in the* 1 to 6 years* group for the nonoperated limb. Higher values for the nonoperated limb in propulsion peak (*p* = 0.043) were found in the* 6 to 11 years* group. There were no statistically significant differences between operated and nonoperated limbs in the* over 11 years* group.

### 3.2. Comparisons between Groups

#### 3.2.1. Operated Limb

The* 1 to 6 years* group had a longer stance phase duration (*p* = 0.008, large effect) compared to the* 6 to 11 years* group. Effect size assessment showed differences between all groups (medium effect). Propulsion peak was significantly higher in the* over 11 years* group compared to the* 6 to 11 years* group (*p* = 0.038, large effect) and to the* 1 to 6 group* (no statistical significance, medium effect). No significant values for the operated limb were found between the* 1 to 6 years* and* over 11 years* groups.

#### 3.2.2. Nonoperated Limb

Comparisons between groups for the nonoperated limb revealed higher values in force and time values. Minimal vertical force was significantly higher in the group* 1 to 6 years* compared to the* 6 to 11 years* group (*p* = 0.031, large effect) and to the* over 11 years* group (no statistical significance, medium effect). Time of minimal vertical force (*p* = 0.023, large effect) was significantly higher in the* over 11 years* group compared to the* 1 to 6 years* group. No significant difference was found between the* 6 to 11 years* and* over 11 years* groups for the nonoperated limb.

Significant asymmetry was found only for the first vertical peak. It means that the first vertical peak occurs earlier on the operated limb compared to the nonoperated limb in the 6 to 11 years group, while in the over 11 years group the first vertical peak occurs earlier on the nonoperated limb. Effect size assessment also showed medium effect on asymmetry, however, only for the temporal variables.

## 4. Discussion

The number of THA revisions is increasing, but few studies investigated the loading symmetry of reoperated limbs or the type and scope of difficulties, especially after longer periods following reoperations. One reason may be that many authors perceive primary and revision operated hips as the same problem with the same features and clinical manifestations. However, other authors note that the difficulties and complications are not the same. Stevens et al. [12] stated that revised hip joints exhibit more limited activities of daily living (ADL), but physical activity of operated individuals is comparable in patients after primary THA. Boonstra et al. [1] found no kinematic or kinetic differences between primary and revision unilateral THA in the sitting to standing test. THA revisions have a structural disadvantage [1, 13], but the newly operated joint may provide some advantages compared to the primary THA. Therefore, we compared the results of our study with the studies of primary unilateral THA surgery based on assumptions of certain similarities in clinical manifestations. However, some differences between primary and reoperated THA are expected.

We found a significant difference during weight acceptance between the reoperated and nonoperated lower limbs during the first years after surgery (*1 to 6 years* after surgery). The nonoperated lower limb reached the first vertical peak earlier in comparison with the operated limb, and the magnitude of the peak was greater. The operated limb came into contact with the floor slowly and with less penetration. McCrory et al. [4] confirmed our results and demonstrated the characteristics of antalgic gait one year after old THA. Our results demonstrated that these characteristics may persist more than one year after surgery. The estimated time of the return to original gait performance is 12 to 18 months after surgery [6–9]. However, these studies and results largely rely on subjective patient assessments.

In the group of* 6 to 11 years* after surgery, results demonstrate the differences in the “propulsion phase” as greater propulsion peak of GRF (in the anterior-posterior direction) for nonoperated limb. It suggests different compensation strategy over the years after surgery. In the first period (5 years after revision), we can attribute it to fear of fall and uncertainty of weight acceptance by the operated limb. In case of more than 6 years since THA revision, the results suggest rather limitation of foot propulsion in the operated limb. The nonoperated limb exhibited more dynamic loading in the anterior-posterior direction. This idea, more active propulsion in anterior-posterior direction for nonoperated limb, is supported also by other clinical studies [21].

After a longer period since THA revision (*over 11 years*) we found no differences in operated and nonoperated lower limbs loading; thus, the lower limbs loading is comparable.

In our study, we also evaluated the level of asymmetry between the limbs. Some scientific studies suggest that asymmetry up to 10% of body weight is to be considered as physiological [22]. Other authors [23] suggest that the upper and lower limits of normal gait asymmetry are variable specific. Therefore, we could consider asymmetry limit value of about 5% for the stance phase duration, about 10% for the force and temporal variables in the vertical direction (with the exception of the time of first vertical peak) and time of propulsion peak, about 20% for braking and propulsion peak, and about 30% for the time of breaking peak. Our results showed mean value of asymmetry higher than 10% only for breaking and propulsion peaks in the group of* 6 to 11 years* after THA revision. We can, therefore, infer that these values are acceptable. A comparison of the groups showed significant differences only in time variables, suggesting possible changes in asymmetry in walking performance compared to pertaining to limbs loading during walking.

Comparisons of the loading of the* operated* lower limb between the 1–6 years and 6–11 years after reoperation groups demonstrated that time after surgery significantly decreased the total time of the stance phase (stance phase duration). It could mean gradual improvement in the timing and general motor control of the operated limb [24]. Comparison of the 6–11 years and over 11 years groups demonstrated increased force during the propulsion phase in the operated lower limb with increasing postoperative duration. Significantly greater dynamics were found in the over 11 years group in the second half of the gait cycle. We can conclude that operated limb starts to gain confidence during reflection, which reduced demands on the reflection of the nonoperated lower limb. These findings are significant in terms of the necessity to produce sufficient level of movement symmetry because of physiological movement stereotypes to avoid overloading of the contralateral joints (not only the hip).

Comparisons of the nonoperated lower limbs between the 1–6 years after surgery and 6–11 years after surgery groups demonstrated higher minimal vertical force in the 1 to 6 years group. Therefore, a natural use of the nonoperated lower limb developed with improved confidence in walking support. However, it is necessary to consider the risk that excessive use of the nonoperated lower limb during gait may cause overloading that accelerates osteoarthritis formation [6, 20]. The nonoperated limb also had significantly protracted time of minimal vertical force in the group of over 11 years in comparison with the 1 to 6 years group. These results suggest the highest asymmetry in the loading pattern within the first 6 years after THA revision.

To the main limitations of the study belong the differences in age between the groups. The over 11 years group is the youngest one. This fact could influence the results of this study; however, walking velocity, which in scientific literature is considered as a very important factor influencing ground reaction force during gait, is comparable among the groups. Another limiting factor is the various postoperative conditions despite the same surgical procedure. This condition could be influenced by different rehabilitation care, which is provided usually in the place of residence. We can also mention here the relatively small sample number of participants in each observed groups.

## 5. Conclusions

The study results suggest different compensation strategies for the operated limb over the years since surgery. In the first years after revision, the GRF pattern showed the tendency of more gradual and safe walking pattern during weight acceptance on the operated limb. In the following years after THA revision, the results suggest limitations of foot propulsion. Despite this fact, lower limb loading could be considered as symmetrical for the entire measured period.

## Figures and Tables

**Table 1:** Comparison of measured values of the operated and nonoperated limb between groups.

Variable	Limb	One to 6 years			Six to 11 years			Over 11 years			Significance (*p*)		
		Mean	Mdn	SD	Mean	Mdn	SD	Mean	Mdn	SD	One to 6 yrs versus 6 to 11 yrs	One to 6 yrs versus over 11 yrs	Six to 11 yrs versus over 11 yrs
Breaking peak [%]	O	−12.2	−12.6	1.9	−11.3	−10.0	2.9	−12.4	−11.5	2.3	0.351	1.000	0.383
	N	−11.9	−12.4	2.3	−12.6	−12.3	2.0	−13.0	−13.1	1.8	0.758	0.351	0.535
Propulsion peak [%]	O	13.8	13.1	2.6	13.6	14.3	2.8	16.4	17.0	1.8	1.000	0.055^+^	0.038^**∗**^
	N	13.3	14.9	5.0	15.5	16.0	2.3	16.9	17.5	2.1	0.536	0.210^+^	0.318
First vertical peak [%]	O	92.2	91.5	7.1	97.3	94.0	8.0	95.7	95.6	3.1	0.299	0.299	1.000
	N	101.3	98.5	7.0	98.7	98.2	5.9	97.7	100.5	4.9	0.606	0.606	0.902
Second vertical peak [%]	O	100.1	99.5	11.6	104.1	103.9	5.2	101.7	98.7	8.7	0.470	0.758	0.318
	N	108.4	109.4	10.2	103.7	101.8	8.4	104.8	106.0	4.0	0.351	0.681	0.710
Minimal vertical force [%]	O	83.9	85.6	7.2	82.8	83.4	4.4	81.0	79.4	4.5	0.536	0.252	0.383
	N	85.0	84.9	3.8	80.4	80.5	2.3	81.1	81.6	3.9	0.031^**∗**^	0.055^+^	1.000

Time of breaking peak [%]	O	57.1	57.0	3.9	57.0	55.3	5.8	56.3	57.5	3.7	0.758	0.837	1.000
	N	58.4	54.7	9.3	54.8	54.8	2.9	56.2	56.2	3.0	0.837	0.758	0.456
Time of propulsion peak [%]	O	42.9	43.0	3.9	43.0	44.7	5.8	43.7	42.5	3.7	0.758	0.837	1.000
	N	41.6	45.3	9.3	45.2	45.2	2.9	43.8	43.8	3.0	0.837	0.758	0.456
Time of first vertical peak [%]	O	27.0	26.8	2.2	26.2	26.6	2.5	26.7	27.8	2.0	0.606	0.837	0.710
	N	23.4	25.1	3.5	23.2	23.2	4.2	27.3	26.1	3.8	0.606	0.114^+^	0.165^+^
Time of second vertical peak [%]	O	75.1	74.4	2.9	73.3	72.8	3.2	75.8	74.6	2.7	0.351	0.536	0.209^+^
	N	76.1	75.6	1.6	76.4	76.1	1.8	76.6	75.5	2.8	0.758	0.918	0.620
Time of minimal vertical force [%]	O	47.2	49.3	4.8	45.9	47.4	6.9	49.9	51.2	8.0	0.918	0.210^+^	0.259^+^
	N	45.1	46.0	4.7	47.7	48.5	4.3	50.6	51.0	2.8	0.299	0.023^**∗**^	0.318
Stance phase duration [s]	O	0.763	0.730	0.088	0.653	0.646	0.052	0.725	0.679	0.094	0.008^**∗**^	0.174^+^	0.097^+^
	N	0.769	0.730	0.096	0.683	0.693	0.087	0.715	0.687	0.067	0.091^+^	0.174^+^	0.805

SD: standard deviation, Mdn: median, O: operated limb, N: nonoperated limb, + = 0.3 ≤*r*≤ 0.5 – medium effect, and *∗* = 0.5 ≤*r* – large effect.

**Table 2:** Comparison of symmetry indexes [%] between groups.

Variable	One to 6 years			Six to 11 years			Over 11 years			Significance (*p*)		
	Mean	Mdn	SD	Mean	Mdn	SD	Mean	Mdn	SD	One to 6 yrs versus 6 to 11 yrs	One to 6 yrs versus over 11 yrs	Six to 11 yrs versus over 11 yrs
Breaking peak	0.7	4.1	14.2	14.8	21.6	28.6	4.0	3.2	21.0	0.408	1.000	0.383
Propulsion peak	−8.9	11.7	47.6	13.9	7.3	19.1	3.7	6.5	12.6	0.681	0.758	0.805
First vertical peak	7.9	2.6	16.6	2.8	3.6	6.9	1.9	1.6	2.3	0.867	0.955	0.535
Second vertical peak	6.5	1.6	15.6	0.5	0.1	3.9	3.1	5.1	6.7	0.694	0.867	0.318
Minimal vertical force	0.9	−2.5	9.8	−3.2	−1.9	4.5	−0.6	−3.1	4.8	0.779	0.955	0.456

Time of breaking peak	−1.1	−6.7	21.8	−3.6	−3.0	11.1	0.2	2.2	8.8	0.758	0.758	0.710
Time of propulsion peak	−2.6	8.9	30.2	5.6	3.2	14.5	0.2	−3.1	10.5	0.681	0.758	0.620
Time of first vertical peak	−6.5	−5.4	19.2	−15.4	−16.1	15.1	7.4	3.9	18.1	0.189^+^	0.397	0.053^*∗*^
Time of second vertical peak	−1.0	−1.4	6.8	1.4	2.1	16.4	6.5	−0.8	20.3	0.613	0.867	0.902
Time of minimal vertical force	0.2	0.9	5.0	4.2	0.9	5.8	1.0	1.0	4.7	0.232^+^	0.867	0.535
Stance phase duration	0.5	0.7	1.9	4.2	6.4	4.2	−1.4	−0.2	6.8	0.091^+^	0.606	0.097^+^

Positive value means higher value of variable on the nonoperated limb and negative value means higher value of variable on the operated limb. Mdn: median, SD: standard deviation, + = 0.1 <*r*< 0.3 – small effect, and *∗* = 0.3 <*r*< 0.5 – medium effect and 0.5 <*r* – large effect.
